# Assessing Community Based Improved Maternal Neonatal Child Survival (IMNCS) Program in Rural Bangladesh

**DOI:** 10.1371/journal.pone.0136898

**Published:** 2015-09-04

**Authors:** Mahfuzar Rahman, Fatema Tuz Jhohura, Sabuj Kanti Mistry, Tridib Roy Chowdhury, Tanveen Ishaque, Rasheduzzaman Shah, Kaosar Afsana

**Affiliations:** 1 Research and Evaluation Division, BRAC, Dhaka, Bangladesh; 2 Department of Global Health, Save the Children USA, Washington, D.C., United States of America; 3 Health, Nutrition, Population Program, BRAC, Dhaka, Bangladesh; Örebro University, SWEDEN

## Abstract

**Objectives:**

A community based approach before, during and after child birth has been proven effective address the burden of maternal, neonatal and child morbidity and mortality in the low and middle income countries. We aimed to examine the overall change in maternal and newborn health outcomes due the “Improved Maternal Newborn and Child Survival” (IMNCS) project, which was implemented by BRAC in rural communities of Bangladesh.

**Methods:**

The intervention was implemented in four districts for duration of 5-years, while two districts served as comparison areas. The intervention was delivered by community health workers who were trained on essential maternal, neonatal and child health care services. A baseline survey was conducted in 2008 among 7, 200 women with pregnancy outcome in last year or having a currently alive child of 12–59 months. A follow-up survey was administered in 2012–13 among 4, 800 women of similar characteristics in the same villages.

**Findings:**

We observed significant improvements in maternal and essential newborn care in intervention areas over time, especially in health care seeking behaviors. The proportion of births taking place at home declined in the intervention districts from 84.3% at baseline to 71.2% at end line (P<0.001). Proportion of deliveries with skilled attendant was higher in intervention districts (28%) compared to comparison districts (27.4%). The number of deliveries was almost doubled at public sector facility comparing with baseline (P<0.001). Significant improvement was also observed in healthy cord care practice, delayed bathing of the new-born and reduction of infant mortality in intervention districts compared to that of comparison districts.

**Conclusions:**

This study demonstrates that community-based efforts offer encouraging evidence and value for combining maternal, neonatal and child health care package. This approach might be considered at larger scale in similar settings with limited resources.

## Introduction

Bangladesh is the 8^th^ populous country in the word [1]. The recent economic growth of Bangladeshis outstanding with a GDP per capita of US$ 1014. The country has made significant progress in improving the health of its population, and has become one of the few countries to be on track to achieve the primary target of Millennium Development Goal (MDG) 5 by 2015 [2]. According to the first MDG progress report, the maternal mortality ratio (MMR) in 1990 was 574 per 100,000 live births in Bangladesh [3, 4]. However, the maternal mortality has declined to 322 in 2001 and 194 in 2010, a 40% decline in nine years as found in Bangladesh Maternal Mortality and Health Care Survey [5]. Health indicators also currently reflect 33.1 infant deaths per 1,000 live births in Bangladesh [4].

The project for improving maternal neonatal and child survival (IMNCS) was designed to improve health outcomes of maternal, neonatal and child survival and was implemented by BRAC’s frontline community health workers. BRAC, one of the largest NGOs in the world, has nearly 40 years of experience in implementing community-based health interventions encompassing rural and more recently urban settings. In 2008, BRAC launched a comprehensive five-year long IMNCS program in rural areas. Intensifying awareness about IMNCS issues were the key components of the IMNCS program. These essential healthcare interventions were provided through an extensive network of local resident women who were trained as community health workers called the *Shasthya Shebika* (*SS*). The IMNCS program was piloted in 2005 in one district; expanded to three more districts in 2008 and six more districts in 2010. The components of IMNCS program included improving awareness on family planning, identification of pregnancies in the early stage, promoting birth planning, providing ANC for pregnant women, delivery care and PNC for recently delivered women, providing essential neonatal care (ENC), management of neonatal illnesses, management of under-5 child illnesses, promoting vaccination, referral for complications of mothers and children and improving the access to clinical services in health facilities.

We assessed the IMNCS programme of BRAC by analyzing the situation for key IMNCS indicators during 2008–2013 in the intervention districts at different stages of project implementation relative with the comparison districts. We examined the overall change due to the project intervention, implemented at the village level, on pregnancy, delivery, postpartum care, and newborn survival compared to same indicators in the comparison areas.

## Methods

### Ethics statement

The ethical approval was obtained from the Bangladesh Medical Research Council (BMRC). The purpose of the study was described to the participants. Both verbal and written consent were provided by participants prior to the baseline and end line interview and the respondents were ensured of the confidentiality of information provided.

### Improving Maternal Neonatal and Child Survival (IMNCS) Program

IMNCS staff and local partners, in consultation with key stakeholders, jointly developed a package of core activities and messages focused on improving home care, increasing care-seeking behaviours, and strengthening community systems related to maternal and new born health services. Table 1 shows the curriculum of community field workers (*SS*, *SK*). The intervention was implemented in 10 districts (out of 64 in the country), selected on the basis of socioeconomic need and low institutional delivery. It was initially implemented by BRAC, UNICEF and Government of Bangladesh.

**Table 1:** Curriculum of the *Sasthya Shebika* (*SS*) and *Sasthya Kormi* (*SK*) training program.

Health workers	Components
*SS*	1. Family planning
	2. Pregnancy, delivery and postpartum care
	3. Essential newborn care
	4. Maternal and neonatal danger sign and complications
	5. Management of birth asphyxia
	6. Special care for low birth weight babies
	7. Neonatal sepsis
	8. Maternal and child nutrition (Infant and Young Child Feeding Practice)
	9. Management of breastfeeding related problems
	10. Vaccination for pregnant woman and children
	11. Identification and management of ten common illness (anaemia, ARI, diarrhoea, dysentery, peptic ulcer, goitre, helminths infestation, skin disease-ring warm and scabies, oral thrush, Night blindness)
	12. Tuberculosis—identification and DOTS
	13. Water and sanitation
	14. Personal hygiene
	15. First aid
*SK*	16. Family planning
	17. Human body, female reproductive system and menstrual cycle
	18. Pregnancy, delivery and postpartum care
	19. Antenatal and postnatal visits and follow-up visits for children under five
	20. Identification and preliminary management of common maternal problems and complications
	21. Measurement of birth weight
	22. Newborn and childcare
	23. Maternal and neonatal danger sign
	24. Management of birth asphyxia
	25. Special care for low birth weight babies
	26. Neonatal sepsis
	27. Maternal and child nutrition (Infant and Young Child Feeding Practice)
	28. Management of breastfeeding related problems
	29. Vaccination for pregnant woman and children
	30. Identification and management of ten common illness (anaemia, ARI, diarrhoea, dysentery, peptic ulcer, goitre, helminths infestation, skin disease-ring warm and scabies, oral thrush, Night blindness)
	31. Tuberculosis—identification and DOTS
	32. Water and sanitation
	33. Personal hygiene
	34. First aid

The comprehensive IMNCS package is designed to address the bottlenecks of demand and supply to ensure a continuum of care from home to hospital. The intervention package is detailed in Table 2. About 19.4 million people living in rural areas are being reached with maternal, neonatal and child health services. The main objectives of IMNCS intervention were: 1) to increase knowledge and practices related to maternal, neonatal and child health, 2) to improve provision of quality maternal, neonatal and child health services at the household and community level, 3) to increase availability and access to quality of maternal, neonatal and child health care and services at facilities, 4) to increase participation, accountability and responsiveness to communities’ voice in maternal, neonatal and child health services. Women were eligible to receive this intervention from the very beginning of their pregnancy until their child was 5 years old. The key indicators for intervention and comparison districts are detailed in Table 3. A bunch of dedicated community health workers namely *Shasthya Shabikas* (*SSs*) and *Shasthya Kormis* (*SKs*) delivered the intervention at the field level while Program Organizers and Managers supervised and provided training to the community health workers. The community health workers provide wide range of domiciliary health services in the communities which include giving messages on family planning and distributing family planning methods, identifying pregnancy, providing ANC, attending delivery and provide immediate newborn care detecting and referring for maternal complications. Also they detect, manage and refer newborn for birth asphyxia, neonatal sepsis, acute respiratory infections (ARI) and provide special care for low birth weight (LBW) babies. The *SSs* and *SKs* visit households six days per week. One *SS* is responsible for 150 households and one SK is responsible for 1500 household and one *SK* supervises 10 *SSs*.

**Table 2:** IMNCS intervention package description.

Program packages	*Intervention districts*	*Comparison districts*
*Community based MNCH committee*		
Formation of village based MNCH committees	Yes	No
*Training of TBAs and linkages with CHWs*		
Training of TBAs on safe deliveries and ties with CHWs	Yes	No
*Program deliveries and training*		
Promotion of antenatal and postnatal care practices	Yes	Yes
Receipt of tetanus toxoid during pregnancy	Yes	Yes
Promotion of birth planning	Yes	No
CHWs’ attendance at delivery	Yes	No
Counseling and communication strategies	Yes	Yes
Effectivereferralsystem^1^	Yes	No
Recognition of complicated pregnancies and sick newborns through maternal and neonatal danger signs	Yes	No
Promotion of adequate maternal nutrition and rest	Yes	Yes
Immediate newborn care including cord care	Yes	No
Promotion of exclusive breastfeeding	Yes	Yes
Ensuring colostrum feeding and early initiation of breastfeeding (within 1 hour of birth)	Yes	No
Promotion of complementary feeding	Yes	No
Promotion of delayed bathing (after 6 hours of delivery)	Yes	Yes

MNCH = Maternal, Neonatal and Child Health; TBA = Traditional Birth Attendant; CHW = Community Health Worker;

^1^Effective referral system = prompt referral, arranged transport, ensure timely services in facility

**Table 3:** Outcome and confounding variables used in evaluation measurement.

Variable	Measurement
	Dependent variables: health outcomes
Stillbirth rate	Total number of child delivered at 7 months of pregnancy or later who didn’t show any signs of life per 1000 birthsinlast year
Infant mortality rate	Total number of infant (<1 year of age) deaths per thousand live births in last year
Maternal danger signs	Faced any one of the complications such as excessive bleeding, convulsion, prolong labor, edema in hand and feet/ severe headache/blurred vision and high fever with foul smelling discharge during last pregnancy
	Dependent variables: accessing care
Ante-natal care (ANC)	Receipt of 4 or more ANC visits in last pregnancy by any provider
TT injection	Receipt of any tetanus toxoids (TT) during last pregnancy or completed dose in prior
Facility delivery	Delivery in government/private health facility
Assisted delivery	Presence of BRAC community health worker (trained traditional birth attendant) during last delivery
Family planning	Using any family planning method (traditional or modern methods)
Birth plan	Having all three major birth plans (delivery place, attendant at delivery and saving money for delivery expenses); procurement of clean delivery kit before delivery
Clean cord care	Cutting cord with sterile blade and tying with sterile thread
Maternal and newborn care	Bathing baby within 6 hours of delivery; Postnatal care (PNC) visit (within 48 hours of delivery) by BRAC community health worker
Breastfeeding practices	Colostrum feeding and initiation of breastfeeding immediately (within 1 hour) after delivery; exclusive breastfeeding (not a single drop of water except breast milk up to 6 months of age)
Demography	Village, union, *upazila*
Socioeconomic	
House	Living in furnished house (brick/cement floor and wall materials)
Fuel	Using wood for cooking purpose
Toilet	Functional sanitary latrine (ring slab with water seal and septic tank)
Personal hygiene	Use of soap for hand wash after defecation
Water	Access to tube well water for drinking

### Shasthya Shebika (*SS*)

*Shasthya Shebika* (*SS*), the frontline community health workers, was selected from the community, aged between 25 to 40 years, married and with at least primary level education. Within 12 months of starting the IMNCS program, BRAC provided all *SS(s)* with their 15 day basic training which included training on family planning, pregnancy identification, and antenatal checkup, assistance on delivery, postnatal checkup, neonatal care and care of under five children. After this basic training, every *SS* had to attend a monthly organized one-day refresher training which helped retaining their knowledge and skill.

### Shasthya Kormi (*SK*)

*Shasthya Kormi* (*SK*), the second level frontline community health workers were the residents of respective area, 20–35 years old and completed secondary education. While *SS* counseled women and mothers, it was the *SK*s who actually provided the ANC, PNC and child care services to the women and mothers within the study areas. The *SKs* received a basic residential training of 18 days which covered identifying pregnant woman, ANC, screening for pregnancy complication, counseling for facility delivery, offering immediate and essential neonatal care, treating ten common illnesses, identifying TB patients and providing DOTS for them, recording data for monthly and annual report preparation (Table 1). The training was also focused on supervisory skill development. Likewise the *SS(s)*, the *SK(s)* attended a refreshers training once every months.

### Study design

This quasi-experimental study was conducted during 2008–2013. Four northern rural districts of Bangladesh were selected for intervention where as two districts were selected for comparison. A basic health care package of BRAC namely Essential Health Care (EHC) was present in both intervention and comparison districts, but IMNCS intervention was absent in comparison areas (Table 2). Selection of comparison districts was based on the geographical and cultural similarities to the intervention districts. The sampling strategy for this survey was a multistage random sampling procedure consisting of selection of *upazilas*, unions, villages and households from selected districts in successive stages.

**Fig 1:**
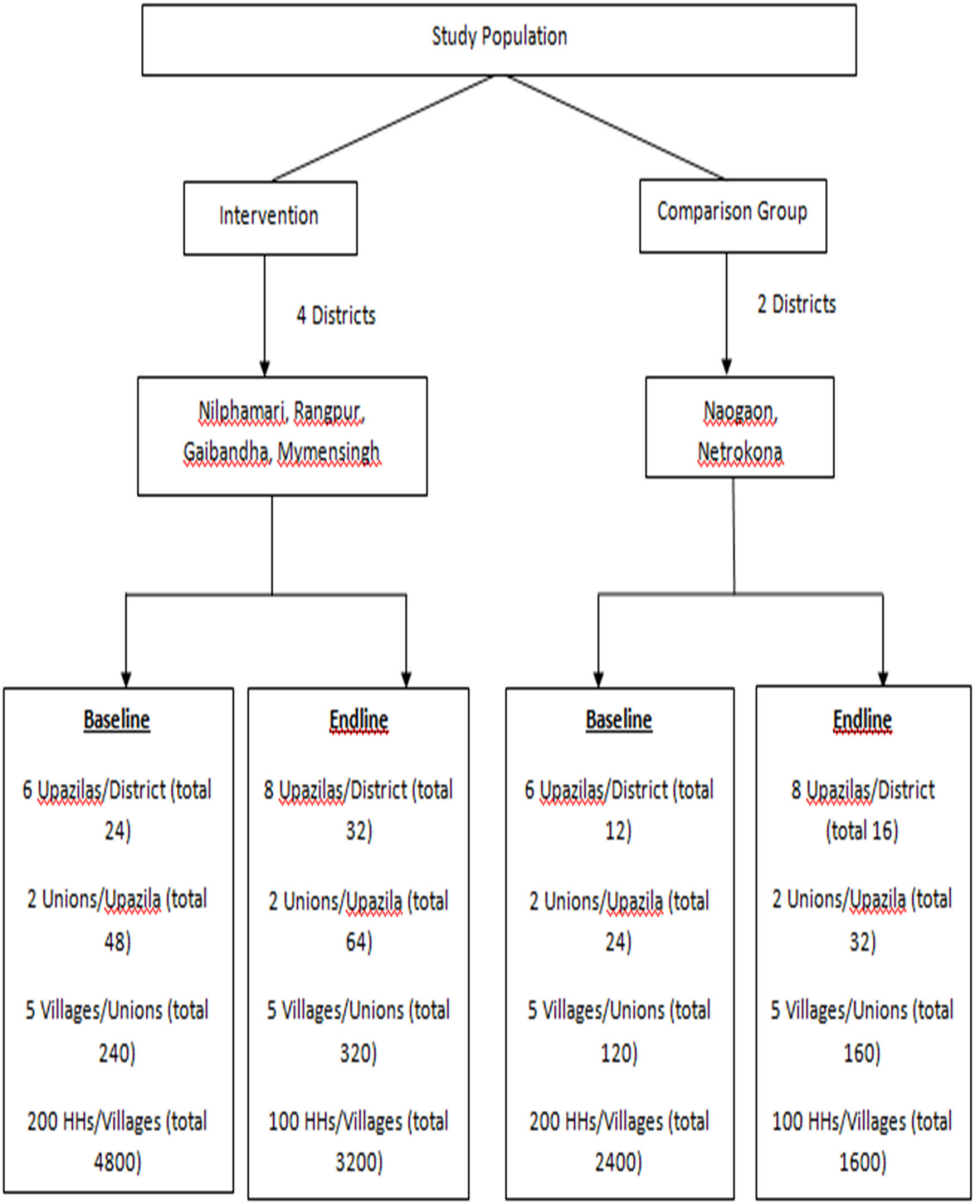
Study flow chart.

Six *upazilas* for baseline and eight *upazilas* for end line were randomly selected from each district; two unions were then selected randomly from each *upazila* and five villages were randomly chosen from each union. Therefore, we have selected 4800 households in intervention areas and 2400 households in comparison areas. In each village, household census was done to have a sampling frame of eligible women for the selection of respondents. Thus, sample of 1200 and 800 per district for baseline and end line was selected respectively. Finally the sample size for the baseline was 7200 and for end-line was 4800. The total sampling procedure is summarized in Fig 1.

The data was collected interviewing the respondents using a pretested structured questionnaire recruiting some interviewers, selected based on prior experience in health survey data collection. A 10 day training for both the surveys was conducted which consisted of lectures and role play to facilitate interview skills. Female interviewers were recruited to ensure maximum participation of the respondents. Towards the end of the training course, the participants spent two days in field practice followed by review and evaluation. The interviewers were divided into number of groups, each headed by a male team leader to supervise the data collection process and to ensure the data quality. Moreover, researchers at the head office monitored field activities through field visits at regular intervals. All interviewers and team leaders were trained by experienced researchers before data collection.

The questionnaire used in the surveys was designed to collect information on the background characteristics of respondents, reproductive history, maternity care information which includes antenatal, delivery and postnatal care and also on complications and treatment-seeking behavior during antenatal, delivery and postnatal period. Newborn care, complications and their treatment-seeking behavior and vaccinations and childhood illnesses and treatment-seeking behavior were also collected. Both the 2008 and 2012 survey questionnaire was pre-tested and revised based upon feedback received in the field test. Seventy enumerators and six monitors were recruited for household listing and surveys. The data were collected during October-January or both the years.

To ensure the quality of data collection, a tight monitoring system was developed for each district. The work of the interviewers was cross-checked by the six rotating monitors who interchanged their places at required intervals. Moreover, the whole field activities were controlled and monitored by a field supervisor. The researchers at the head office monitored field activities through field visits at regular intervals. Intensive supervision, on-the-spot checking for inconsistencies, and a random post-enumeration survey of 5% of the households surveyed in the 72 hours were carried to identify data discrepancy. The respondents were re interviewed if any data discrepancy was identified.

### Statistical Analysis

Data analysis included summary statistics and descriptive statistics used in comparing the grouped districts. Descriptive statistics were used to observe whether the intervention districts achieved the target, set for certain MNCH related key indicators. Univariate analysis was carried out to assess the relationship between key individual IMNCS indicators and specific independent variables. Univariate analyses involved chi-square tests for calculating differences of proportions between groups over the period of intervention. Data analyses were performed by taking into account the study design.

## Results

Our project covered 14 districts, 93 upozillas, encompassing a population of 7.1 million. This intervention knowledge has been employed by community health workers visiting every hou*se*hold in their assigned and catchment area, which means covering100% of population in respective intervention districts. Table 4 (Table A in S1 Supporting Information) shows the baseline demographic and socioeconomic characteristics of intervention and comparison districts and details of births and infant deaths. On average, intervention districts have more wood dust for cooking (29.0%*vs*. 23.0%) and sanitary latrine (15.6%*vs*. 12.0%), but lower electricity than comparison (26.1%*vs*. 30.7%). Overall live births rates were comparable, but the average still birth rate at baseline was slightly higher in comparison districts than intervention (10.9% *vs*. 13.5%). Intervention districts have slightly higher infant mortality rate than comparison (45.9% *vs*. 40.3%). However, the findings are showing some encouraging impact which ultimately would help to extend the program in other lower impacted areas (Table 5, Table B in S1 Supporting Information).

**Table 4:** Baseline characteristics of the intervention and comparison districts, October 2008-January 2009.

Characteristics	Intervention district					Comparison districts		
	Nil	Rang	Gai	Mymen	Total	Naog	Netro	Total
Demographic characteristics								
Upazila	6	6	6	6	24	6	6	12
Union	12	12	12	12	48	12	12	24
Village	60	60	60	60	240	60	60	120
Households	1200	1200	1200	1200	4800	1200	1200	2400
Women of reproductive age	1200	1200	1200	1200	4800	1200	1200	2400
Pregnant women	57	46	45	75	223	33	86	119
Live births (last one year)	539	484	514	555	2092	496	530	1026
Still births(last one year)	5	7	4	7	23	8	6	14
Total births (last one year)	544	491	518	562	2115	504	536	1040
Infant death	28	23	20	26	97	12	30	42
Stillbirth rate (per 1000 births)	9.3	14.5	7.8	12.6	10.87	16.1	11.3	13.46
Infant mortality rate (per 1000 live births)	51.9	47.5	38.9	46.8	45.86	24.2	56.6	40.38
Socioeconomic indicators									
Households with electricity (%)	20.7	29.4	20.4	33.9	26.1	41.8	19.7	30.7
Pucca (brick/cement) floor and wall materials	3.4	5.8	3.8	2.8	4.0	6.8	2.2	4.5
Wood used for cooking (%)	23.1	19.7	21.3	54.3	29.6	9.9	37.6	23.8
Functional sanitary latrine (%)	23.2	13.4	13.9	11.8	15.6	11.8	12.2	12.0
Used tube well for drinking water	97.9	98.1	99.7	97.8	98.4	97.0	99.3	98.2
Used Soap after defecation	56.2	55.2	39.1	41.3	48.0	60.3	39.2	49.8

**Table 5:** Birth and mortality data following the intervention, October 2012-January 2013.

Characteristics	Intervention district					Comparison districts		
	Nil	Rang	Gai	Mymen	Total	Naog	Netro	Total
Upazila	8	8	8	8	32	8	8	16
Union	16	16	16	16	64	16	16	32
Village	80	80	80	80	320	80	80	160
Households	800	800	800	800	3200	800	800	1600
Women of reproductive age	800	800	800	800	3200	800	800	1600
Pregnant women	34	24	22	42	122	28	39	67
Live births (last one year)	328	332	344	354	1358	310	361	671
Still births(last one year)	3	4	6	6	19	3	7	10
Total births (last one year)	331	336	350	360	1377	313	368	681
Infant death	21	9	12	20	62	9	14	23
Stillbirth rate (per 1000 births)^a^	9.06 (-2.58)	11.90 (-17.93)	17.14 (119.74)	16.67 (32.30)	13.80 (26.95)	9.58 (-40.50)	19.02 (68.32)	14.68 (9.06)
Infant mortality rate (per 1000 live births)^a^	63.44 (22.24)	26.79 (-43.60)	34.29 (-11.85)	55.56 (18.72)	45.03 (-1.81)	28.75 (18.80)	38.04 (-32.79)	33.77 (-16.37)

^a^ Value in parentheses is the percentage change from baseline

Intervention districts demonstrated important differences especially in cutting and caring of cord with sterile materials (P<0.001). Proportion of deliveries with skilled attendant was higher in intervention districts (28%) compared to comparison districts (27.4%). In terms of delayed bathing baby (bathing baby after 6 hours of birth) higher percentage have observed in intervention districts than that of comparison districts, seemingly meaning of adequate knowledge being disseminated in the intervention areas. Exclusive breast feeding (EBF) practice was found greater in intervention districts than comparison areas (34.5% vs. 26.4%; P<.0.001). However, according to the national survey the level of exclusive breastfeeding among children under six months, 64 percent in 2011. The survey collected data on infant feeding for the youngest children less than two years who are living with their mother using a 24-hour recall period. Part of the increase (about 4 percentage points) is explained by a change in the age pattern in the sample with a higher proportion of infants 0–3 months in the 2011. Additionally, there is the possible effect of several intensive programs that focus on maternal and new-born care and child health, including improved feeding, that has been implemented for the 1–2 years before the survey and cover about 25 percent of the country's population. One of the plausible explanation for exclusive breast feeding rate found lower in both in intervention and control area may be due to using 6 month instead of 24 hour recall period during interview might be responsible for comparatively lower percentage of EBF both in intervention and control district.

Furthermore respondents’ knowledge regarding post-natal care (PNC) was significantly higher in intervention districts than comparison districts (P<0.001). Community base health workers have visited every household for identifying each pregnancy and thereafter ensuring their pre- and post-natal visits, appropriately referred and counselled for assisted delivery at home or promotion of facility based delivery either at government or private facility (Table 6, Table B in S1 Supporting Information).

**Table 6:** Perinatal care and care provision reported after the intervention in randomly selected intervention and comparison district.

Characteristics	Sample of intervention district		Sample of comparison district		
	n	%	n	%	P value
Women who have any outcome last one year or have under five child	3200	100	1600	100	
Ever had any abortion	441	13.8	246	15.4	0.567
Ever had any MR	123	3.8	48	3.0	0.800
Currently used family planning method	2208	69.0	1056	66.0	0.086
Currently using any modern FP method	2042	63.8	978	61.1	0.151
Mother who have any outcome last one year	1600	100	800	100	
Pregnancy identified by %					
Self	798	49.9	508	63.5	0.000
BRAC CHW^a^	370	23.1	14	1.8	0.000
Other^b^	266	16.6	170	21.2	0.006
Antenatal care check-up during last pregnancy	1427	89.2	553	69.1	0.000
Having 3–5 knowledge on maternal danger sign	703	44.0	226	28.2	0.000
Proportion who face maternal danger sign during last delivery	678	42.4	493	61.6	0.000
Receipt of tetanus toxoid during pregnancy	806	50.4	319	39.9	0.000
Delivery in government health facility	228	14.2	82	10.2	0.006
Women having all 3 birth plans (place, attendant, money)	645	40.3	282	35.2	0.016
Procurement of clean delivery kit before delivery	602	37.6	159	19.9	0.000
Delivery by skilled birth attendant	448	28.0	219	27.4	0.853
Presence of BRAC CHW during last delivery	459	28.7	1	0.1	0.000
Mother who have delivered live birth	1358	100	671	100	
Cutting and tying cord with sterile materials	1271	93.6	574	85.5	0.000
Bathing the baby within 6 hours of birth	133	9.8	78	11.6	0.211
Colostrum and breastfeeding within an hour of birth	1081	79.6	527	78.5	0.565
Newborn was exclusively breastfed	468	34.5	177	26.4	0.000
Postnatal visit within 48 hours by trained provider^c^	1091	80.3	198	29.5	0.000

^a^ CHW = community health workers

^b^ Husband, WV, FWA, Doctor, Nurse, TBA, village doctor, etc

^c^ Qualified doctor, FWV, nurse, midwife, paramedic, BRAC SK

**Fig 2:**
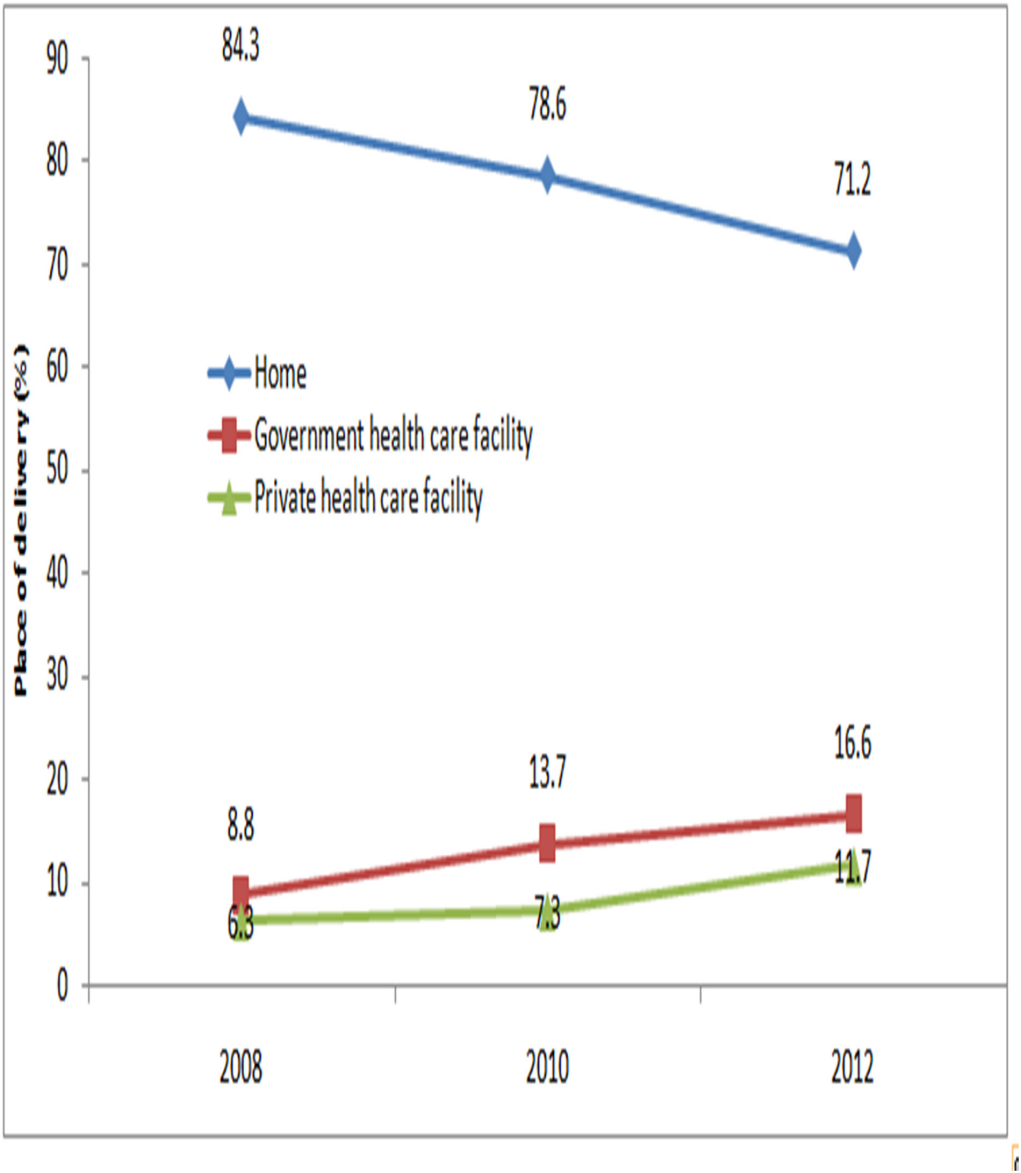
Change in place of delivery for women from intervention districts, during the study.

The intervention districts showed that the proportion of home deliveries declined in the intervention districts from 84.3% at baseline to 71.2% at end line (Fig 2, Table C in S1 Supporting Information) (P<0.001). In contrary, facility deliveries doubled from 8.8% to 16.6% in government facility (P<0.001). Public sector facility delivery was also concurrently rose significantly from 6.3% to 11.7% (P<0.001).

## Discussion

The results suggested a noteworthy improvement in intervention districts than comparison districts, specially focusing on increasing facility delivery, skilled attendant at delivery, maternal care during pregnancy, post pregnancy, neonatal and infant care. Our study is the first in Bangladesh covering a large population of around 20 million in rural areas.

The survey population shows a notable achievement regarding ANC practices in intervention districts. The proportion of those receiving recommended four or more ANC visits accounted average 89% by 2012 in the intervention districts which is much higher than comparison districts, as well as sharp increase from baseline (14.8% in 2008). The increase in four ANC may be attributable to the door-to-door free services provided by the BRAC SKs. Similar finding was noted in a study conducted in Matlab, Bangladesh where services were delivered by community health research worker at home or outreach clinic [6, 7]. Other studies also reported the increasing trend in ANC practice, while the proportion was comparatively lower [4, 8]. The difference was probably due to *SS*s motivating the pregnant women to receive ANC and *SK*s delivering the ANC services to the doorsteps. Studies have showed that the ability to fully utilize ANC services is affected by availability, accessibility and quality of services, women’s socio economic and education status, demographic factors, cultural beliefs and previous obstetric history [9–12]. ANC visits helps in diagnosis of pregnancy complications and their treatment regimens [13], motivating for facility delivery and improving perinatal survival [7].

Delivery with skilled birth attendant (SBA) is one of the main proxy indicators globally adopted to monitor progress in MDG-5 [14]. Several studies have presented evidence of a positive association between SBA utilization during childbirth and reduction in maternal mortality [15–19]. A comparatively higher skilled assistance at childbirth in intervention districts shows a slow but unmistakable movement towards professionalism. The survey results revealed that delivery at home has been the commonest practice throughout the study area though the frequency decreased after intervention. Unavailability of maternal health care services, low socio-economic status, low education, and residence in rural areas results in low use of skilled care by women during childbirth. It might be that some women prefer home birth because they fear to lose social status, confidentiality, and comparison over the birth process with facility delivery [20–22]. In some instances, decision to deliver at a facility doesn’t depend on the woman but on the husbands, mother-in-laws, community heads, soothsayers or traditional healers [23, 24]. Many women prefer and seek to give birth under conditions where they feel safe, protected and secure and in most cases it is home [25, 26]. However, increased level of awareness regarding the safe delivery practices at the community level, prompt referral, arranged transport and proper management in the facility ensured by IMNCS intervention may have caused increased facility delivery compared to that of home deliveries in intervention districts overtime. An increasing trend in facility delivery in intervention areas indicates an increasing percentage of births attended by medically trained providers. This was largely explained by the increase in the proportion of births at which a skilled attendant assisted [27].

BRAC *SS*s advised women to develop a birth plan that ensures birth preparedness and readiness in the eventuality of pregnancy or childbirth complication [28]. Such a birth plan is expected to assist women in making choices that would contribute to a good pregnancy outcome. The key elements of the birth plan package included recognition of danger signs, choosing a birth attendant and place of delivery, saving money for transport and other costs in case of emergency, identification of a potential blood donor and a decision maker [29, 30].

Postnatal care plays a significant role in improving maternal health and preventing maternal and neonatal deaths, especially PNC within 2 postpartum days [20, 31, 32]. The use of skilled attendant at birth should guarantee that care in the immediate postpartum period is available. One-fourth of the women did not receive PNC within two days of delivery from a medically trained provider in Bangladesh, according to a recent survey [33]. In intervention districts 80.3% of the mothers received PNC within 48 hours which was only 29.5% in comparison areas indicating improved performance. According to the Bangladesh Demographic and Health Survey 2011, 27% of the mothers receive PNC within two days and 29% within 42 days of delivery [8].

The results suggested that women’s awareness about maternal danger signs had increased over the years. More women in intervention districts (44%) continued to retain knowledge of three or more (maximum five) compared to other districts (Table 6). There has not been any significant improvement in the proportion of women knowing the BRAC referral number or calling BRAC *SS/SK* for management of maternal danger signs. However, 9 out of 10 of women in all districts knew to seek treatment from the referral places or hospitals when any complications arise (data not shown). Other studies found that irrespective of their needs, only people from higher economic or educational groups can afford to seek healthcare from trained personnel in Bangladesh. It was found that younger mothers were significantly less likely to seek professional healthcare at the time of birth [34].

There were many improvements considering maternal and neonatal healthcare, but the study had several limitations that should be recognized. Firstly, inclusion of the women was conditional upon ever having married and as being or having been a mother (in the case of a child death). Secondly, the groups differed in some important respects; in particular the number of *SS*/*SK* per inhabitant was higher in intervention districts compared to comparison districts. Thirdly, the quasi-experimental nature of the study presents certain limitations as the comparison districts may possess similar other MNCH interventions which might overestimate or underestimate the actual impact. Fourthly, since randomization was followed from sub-district to household level in each district, therefore the result does not represent the urban scenario. Finally, the survey was also subjected to several forms of biases like recall bias, interviewer bias and information bias. However, several strategies like recruiting experienced interviewers, standardized training on questionnaire probing, rapport building with interviewees and intensive supervision were adopted to address the biases

Our data provided evidence that maternal and infant outcomes can be positively influenced by an intervention package delivered through a community care and outreach strategy within the existing health system. However, in our health system, focuses need to be emphasized specially on ensuring ante natal care which can prepare all mothers for their safe delivery and offspring well-being. Further analyses on equity or cost beneficiary or cost-effectiveness issues are necessary, to enlighten policy decision about a national level scaling up successful community based program.

## Supporting Information

S1 Supporting Information Additional description. Table A. Frequency of IMNCS thana list into the selected district.
Table B. Percentage of ever had abortion.
Table C. Distribution of home and facility deliveries.
(DOCX)
