# Supplementary material for: Assessing Community Based Improved Maternal Neonatal Child Survival (IMNCS) Program in Rural Bangladesh
Source: PLoS One. 2015 Sep 4;10(9):e0136898. doi: 10.1371/journal.pone.0136898 (PMC4560389; doi:10.1371/journal.pone.0136898)
Supplement: S1 Supporting Information — Table A. Frequency of IMNCS thana list into the selected district. Table B. Percentage of ever had abortion. Table C. Distribution of home and facility deliveries. (DOCX) [file pone.0136898.s001.docx]

**S1 Supporting information, additional description**

**SA Table. Frequency of IMNCS thana list into the selected district**

| **thana * dist_old_intCrosstabulation** | | | | | | |
| --- | --- | --- | --- | --- | --- | --- |
| Count | | | | | | |
|  | | dist_old_int | | | | Total |
|  |  | 1.00 | 2.00 | 3.00 | 4.00 |  |
| thana | 1 | 200 | 0 | 0 | 0 | 200 |
|  | 2 | 200 | 0 | 0 | 0 | 200 |
|  | 3 | 200 | 0 | 0 | 0 | 200 |
|  | 4 | 200 | 0 | 0 | 0 | 200 |
|  | 5 | 200 | 0 | 0 | 0 | 200 |
|  | 6 | 200 | 0 | 0 | 0 | 200 |
|  | 7 | 0 | 200 | 0 | 0 | 200 |
|  | 8 | 0 | 200 | 0 | 0 | 200 |
|  | 9 | 0 | 201 | 0 | 0 | 201 |
|  | 10 | 0 | 200 | 0 | 0 | 200 |
|  | 11 | 0 | 200 | 0 | 0 | 200 |
|  | 12 | 0 | 200 | 0 | 0 | 200 |
|  | 13 | 0 | 0 | 200 | 0 | 200 |
|  | 14 | 0 | 0 | 200 | 0 | 200 |
|  | 15 | 0 | 0 | 200 | 0 | 200 |
|  | 16 | 0 | 0 | 200 | 0 | 200 |
|  | 17 | 0 | 0 | 200 | 0 | 200 |
|  | 18 | 0 | 0 | 200 | 0 | 200 |
|  | 19 | 0 | 0 | 0 | 200 | 200 |
|  | 20 | 0 | 0 | 0 | 200 | 200 |
|  | 21 | 0 | 0 | 0 | 200 | 200 |
|  | 22 | 0 | 0 | 0 | 200 | 200 |
|  | 23 | 0 | 0 | 0 | 200 | 200 |
|  | 24 | 0 | 0 | 0 | 200 | 200 |
| Total | | 1200 | 1201 | 1200 | 1200 | 4801 |

| **union * dist_old_intCrosstabulation** | | | | | | |
| --- | --- | --- | --- | --- | --- | --- |
| Count | | | | | | |
|  | | dist_old_int | | | | Total |
|  |  | 1.00 | 2.00 | 3.00 | 4.00 |  |
| union | 1 | 100 | 0 | 0 | 0 | 100 |
|  | 2 | 100 | 0 | 0 | 0 | 100 |
|  | 3 | 100 | 0 | 0 | 0 | 100 |
|  | 4 | 100 | 0 | 0 | 0 | 100 |
|  | 5 | 100 | 0 | 0 | 0 | 100 |
|  | 6 | 100 | 0 | 0 | 0 | 100 |
|  | 7 | 100 | 0 | 0 | 0 | 100 |
|  | 8 | 100 | 0 | 0 | 0 | 100 |
|  | 9 | 100 | 0 | 0 | 0 | 100 |
|  | 10 | 100 | 0 | 0 | 0 | 100 |
|  | 11 | 100 | 0 | 0 | 0 | 100 |
|  | 12 | 100 | 0 | 0 | 0 | 100 |
|  | 13 | 0 | 100 | 0 | 0 | 100 |
|  | 14 | 0 | 100 | 0 | 0 | 100 |
|  | 15 | 0 | 100 | 0 | 0 | 100 |
|  | 16 | 0 | 100 | 0 | 0 | 100 |
|  | 17 | 0 | 101 | 0 | 0 | 101 |
|  | 18 | 0 | 100 | 0 | 0 | 100 |
|  | 19 | 0 | 100 | 0 | 0 | 100 |
|  | 20 | 0 | 100 | 0 | 0 | 100 |
|  | 21 | 0 | 100 | 0 | 0 | 100 |
|  | 22 | 0 | 100 | 0 | 0 | 100 |
|  | 23 | 0 | 100 | 0 | 0 | 100 |
|  | 24 | 0 | 100 | 0 | 0 | 100 |
|  | 25 | 0 | 0 | 100 | 0 | 100 |
|  | 26 | 0 | 0 | 100 | 0 | 100 |
|  | 27 | 0 | 0 | 100 | 0 | 100 |
|  | 28 | 0 | 0 | 100 | 0 | 100 |
|  | 29 | 0 | 0 | 100 | 0 | 100 |
|  | 30 | 0 | 0 | 100 | 0 | 100 |
|  | 31 | 0 | 0 | 100 | 0 | 100 |
|  | 32 | 0 | 0 | 100 | 0 | 100 |
|  | 33 | 0 | 0 | 100 | 0 | 100 |
|  | 34 | 0 | 0 | 100 | 0 | 100 |
|  | 35 | 0 | 0 | 100 | 0 | 100 |
|  | 36 | 0 | 0 | 100 | 0 | 100 |
|  | 37 | 0 | 0 | 0 | 100 | 100 |
|  | 38 | 0 | 0 | 0 | 100 | 100 |
|  | 39 | 0 | 0 | 0 | 100 | 100 |
|  | 40 | 0 | 0 | 0 | 100 | 100 |
|  | 41 | 0 | 0 | 0 | 100 | 100 |
|  | 42 | 0 | 0 | 0 | 100 | 100 |
|  | 43 | 0 | 0 | 0 | 100 | 100 |
|  | 44 | 0 | 0 | 0 | 100 | 100 |
|  | 45 | 0 | 0 | 0 | 100 | 100 |
|  | 46 | 0 | 0 | 0 | 100 | 100 |
|  | 47 | 0 | 0 | 0 | 100 | 100 |
|  | 48 | 0 | 0 | 0 | 100 | 100 |
| Total | | 1200 | 1201 | 1200 | 1200 | 4801 |

| **villg * dist_old_intCrosstabulation** | | | | | | |
| --- | --- | --- | --- | --- | --- | --- |
| Count | | | | | | |
|  | | dist_old_int | | | | Total |
|  |  | 1.00 | 2.00 | 3.00 | 4.00 |  |
| villg | 1 | 0 | 0 | 0 | 20 | 20 |
|  | 2 | 0 | 0 | 0 | 20 | 20 |
|  | 3 | 0 | 0 | 0 | 20 | 20 |
|  | 4 | 0 | 0 | 0 | 20 | 20 |
|  | 5 | 0 | 0 | 0 | 20 | 20 |
|  | 6 | 0 | 0 | 0 | 20 | 20 |
|  | 7 | 0 | 0 | 0 | 20 | 20 |
|  | 8 | 0 | 0 | 0 | 20 | 20 |
|  | 9 | 0 | 0 | 0 | 20 | 20 |
|  | 10 | 0 | 0 | 0 | 20 | 20 |
|  | 11 | 0 | 0 | 0 | 20 | 20 |
|  | 12 | 0 | 0 | 0 | 20 | 20 |
|  | 13 | 0 | 0 | 0 | 20 | 20 |
|  | 14 | 0 | 0 | 0 | 20 | 20 |
|  | 15 | 0 | 0 | 0 | 20 | 20 |
|  | 16 | 0 | 0 | 0 | 20 | 20 |
|  | 17 | 0 | 0 | 0 | 20 | 20 |
|  | 18 | 0 | 0 | 0 | 20 | 20 |
|  | 19 | 0 | 0 | 0 | 20 | 20 |
|  | 20 | 0 | 0 | 0 | 20 | 20 |
|  | 21 | 0 | 0 | 0 | 20 | 20 |
|  | 22 | 0 | 0 | 0 | 20 | 20 |
|  | 23 | 0 | 0 | 0 | 20 | 20 |
|  | 24 | 0 | 0 | 0 | 20 | 20 |
|  | 25 | 0 | 0 | 0 | 20 | 20 |
|  | 26 | 0 | 0 | 0 | 20 | 20 |
|  | 27 | 0 | 0 | 0 | 20 | 20 |
|  | 28 | 0 | 0 | 0 | 20 | 20 |
|  | 29 | 0 | 0 | 0 | 20 | 20 |
|  | 30 | 0 | 0 | 0 | 20 | 20 |
|  | 61 | 20 | 0 | 0 | 0 | 20 |
|  | 62 | 20 | 0 | 0 | 0 | 20 |
|  | 63 | 20 | 0 | 0 | 0 | 20 |
|  | 64 | 20 | 0 | 0 | 0 | 20 |
|  | 65 | 20 | 0 | 0 | 0 | 20 |
|  | 66 | 20 | 0 | 0 | 0 | 20 |
|  | 67 | 20 | 0 | 0 | 0 | 20 |
|  | 68 | 20 | 0 | 0 | 0 | 20 |
|  | 69 | 20 | 0 | 0 | 0 | 20 |
|  | 70 | 20 | 0 | 0 | 0 | 20 |
|  | 71 | 20 | 0 | 0 | 0 | 20 |
|  | 72 | 20 | 0 | 0 | 0 | 20 |
|  | 73 | 20 | 0 | 0 | 0 | 20 |
|  | 74 | 20 | 0 | 0 | 0 | 20 |
|  | 75 | 20 | 0 | 0 | 0 | 20 |
|  | 76 | 20 | 0 | 0 | 0 | 20 |
|  | 77 | 20 | 0 | 0 | 0 | 20 |
|  | 78 | 20 | 0 | 0 | 0 | 20 |
|  | 79 | 20 | 0 | 0 | 0 | 20 |
|  | 80 | 20 | 0 | 0 | 0 | 20 |
|  | 81 | 20 | 0 | 0 | 0 | 20 |
|  | 82 | 20 | 0 | 0 | 0 | 20 |
|  | 83 | 20 | 0 | 0 | 0 | 20 |
|  | 84 | 20 | 0 | 0 | 0 | 20 |
|  | 85 | 20 | 0 | 0 | 0 | 20 |
|  | 86 | 20 | 0 | 0 | 0 | 20 |
|  | 87 | 20 | 0 | 0 | 0 | 20 |
|  | 88 | 20 | 0 | 0 | 0 | 20 |
|  | 89 | 20 | 0 | 0 | 0 | 20 |
|  | 90 | 20 | 0 | 0 | 0 | 20 |
|  | 91 | 0 | 20 | 0 | 0 | 20 |
|  | 92 | 0 | 20 | 0 | 0 | 20 |
|  | 93 | 0 | 20 | 0 | 0 | 20 |
|  | 94 | 0 | 20 | 0 | 0 | 20 |
|  | 95 | 0 | 20 | 0 | 0 | 20 |
|  | 96 | 0 | 20 | 0 | 0 | 20 |
|  | 97 | 0 | 20 | 0 | 0 | 20 |
|  | 98 | 0 | 20 | 0 | 0 | 20 |
|  | 99 | 0 | 20 | 0 | 0 | 20 |
|  | 100 | 0 | 20 | 0 | 0 | 20 |
|  | 101 | 0 | 20 | 0 | 0 | 20 |
|  | 102 | 0 | 20 | 0 | 0 | 20 |
|  | 103 | 0 | 21 | 0 | 0 | 21 |
|  | 104 | 0 | 20 | 0 | 0 | 20 |
|  | 105 | 0 | 20 | 0 | 0 | 20 |
|  | 106 | 0 | 20 | 0 | 0 | 20 |
|  | 107 | 0 | 20 | 0 | 0 | 20 |
|  | 108 | 0 | 20 | 0 | 0 | 20 |
|  | 109 | 0 | 20 | 0 | 0 | 20 |
|  | 110 | 0 | 20 | 0 | 0 | 20 |
|  | 111 | 0 | 20 | 0 | 0 | 20 |
|  | 112 | 0 | 20 | 0 | 0 | 20 |
|  | 113 | 0 | 20 | 0 | 0 | 20 |
|  | 114 | 0 | 20 | 0 | 0 | 20 |
|  | 115 | 0 | 20 | 0 | 0 | 20 |
|  | 116 | 0 | 20 | 0 | 0 | 20 |
|  | 117 | 0 | 20 | 0 | 0 | 20 |
|  | 118 | 0 | 20 | 0 | 0 | 20 |
|  | 119 | 0 | 20 | 0 | 0 | 20 |
|  | 120 | 0 | 20 | 0 | 0 | 20 |
|  | 151 | 0 | 0 | 20 | 0 | 20 |
|  | 152 | 0 | 0 | 20 | 0 | 20 |
|  | 153 | 0 | 0 | 20 | 0 | 20 |
|  | 154 | 0 | 0 | 20 | 0 | 20 |
|  | 155 | 0 | 0 | 20 | 0 | 20 |
|  | 156 | 0 | 0 | 20 | 0 | 20 |
|  | 157 | 0 | 0 | 20 | 0 | 20 |
|  | 158 | 0 | 0 | 20 | 0 | 20 |
|  | 159 | 0 | 0 | 20 | 0 | 20 |
|  | 160 | 0 | 0 | 20 | 0 | 20 |
|  | 161 | 0 | 0 | 20 | 0 | 20 |
|  | 162 | 0 | 0 | 20 | 0 | 20 |
|  | 163 | 0 | 0 | 20 | 0 | 20 |
|  | 164 | 0 | 0 | 20 | 0 | 20 |
|  | 165 | 0 | 0 | 20 | 0 | 20 |
|  | 166 | 0 | 0 | 20 | 0 | 20 |
|  | 167 | 0 | 0 | 20 | 0 | 20 |
|  | 168 | 0 | 0 | 20 | 0 | 20 |
|  | 169 | 0 | 0 | 20 | 0 | 20 |
|  | 170 | 0 | 0 | 20 | 0 | 20 |
|  | 171 | 0 | 0 | 20 | 0 | 20 |
|  | 172 | 0 | 0 | 20 | 0 | 20 |
|  | 173 | 0 | 0 | 20 | 0 | 20 |
|  | 174 | 0 | 0 | 20 | 0 | 20 |
|  | 175 | 0 | 0 | 20 | 0 | 20 |
|  | 176 | 0 | 0 | 20 | 0 | 20 |
|  | 177 | 0 | 0 | 20 | 0 | 20 |
|  | 178 | 0 | 0 | 20 | 0 | 20 |
|  | 179 | 0 | 0 | 20 | 0 | 20 |
|  | 180 | 0 | 0 | 20 | 0 | 20 |
|  | 181 | 0 | 0 | 20 | 0 | 20 |
|  | 182 | 0 | 0 | 20 | 0 | 20 |
|  | 183 | 0 | 0 | 20 | 0 | 20 |
|  | 184 | 0 | 0 | 20 | 0 | 20 |
|  | 185 | 0 | 0 | 20 | 0 | 20 |
|  | 186 | 0 | 0 | 20 | 0 | 20 |
|  | 187 | 0 | 0 | 20 | 0 | 20 |
|  | 188 | 0 | 0 | 20 | 0 | 20 |
|  | 189 | 0 | 0 | 20 | 0 | 20 |
|  | 190 | 0 | 0 | 20 | 0 | 20 |
|  | 191 | 0 | 0 | 20 | 0 | 20 |
|  | 192 | 0 | 0 | 20 | 0 | 20 |
|  | 193 | 0 | 0 | 20 | 0 | 20 |
|  | 194 | 0 | 0 | 20 | 0 | 20 |
|  | 195 | 0 | 0 | 20 | 0 | 20 |
|  | 196 | 0 | 0 | 20 | 0 | 20 |
|  | 197 | 0 | 0 | 20 | 0 | 20 |
|  | 198 | 0 | 0 | 20 | 0 | 20 |
|  | 199 | 0 | 0 | 20 | 0 | 20 |
|  | 200 | 0 | 0 | 20 | 0 | 20 |
|  | 201 | 0 | 0 | 20 | 0 | 20 |
|  | 202 | 0 | 0 | 20 | 0 | 20 |
|  | 203 | 0 | 0 | 20 | 0 | 20 |
|  | 204 | 0 | 0 | 20 | 0 | 20 |
|  | 205 | 0 | 0 | 20 | 0 | 20 |
|  | 206 | 0 | 0 | 20 | 0 | 20 |
|  | 207 | 0 | 0 | 20 | 0 | 20 |
|  | 208 | 0 | 0 | 20 | 0 | 20 |
|  | 209 | 0 | 0 | 20 | 0 | 20 |
|  | 210 | 0 | 0 | 20 | 0 | 20 |
|  | 211 | 20 | 0 | 0 | 0 | 20 |
|  | 212 | 20 | 0 | 0 | 0 | 20 |
|  | 213 | 20 | 0 | 0 | 0 | 20 |
|  | 214 | 20 | 0 | 0 | 0 | 20 |
|  | 215 | 20 | 0 | 0 | 0 | 20 |
|  | 216 | 20 | 0 | 0 | 0 | 20 |
|  | 217 | 20 | 0 | 0 | 0 | 20 |
|  | 218 | 20 | 0 | 0 | 0 | 20 |
|  | 219 | 20 | 0 | 0 | 0 | 20 |
|  | 220 | 20 | 0 | 0 | 0 | 20 |
|  | 221 | 20 | 0 | 0 | 0 | 20 |
|  | 222 | 20 | 0 | 0 | 0 | 20 |
|  | 223 | 20 | 0 | 0 | 0 | 20 |
|  | 224 | 20 | 0 | 0 | 0 | 20 |
|  | 225 | 20 | 0 | 0 | 0 | 20 |
|  | 226 | 20 | 0 | 0 | 0 | 20 |
|  | 227 | 20 | 0 | 0 | 0 | 20 |
|  | 228 | 20 | 0 | 0 | 0 | 20 |
|  | 229 | 20 | 0 | 0 | 0 | 20 |
|  | 230 | 20 | 0 | 0 | 0 | 20 |
|  | 231 | 20 | 0 | 0 | 0 | 20 |
|  | 232 | 20 | 0 | 0 | 0 | 20 |
|  | 233 | 20 | 0 | 0 | 0 | 20 |
|  | 234 | 20 | 0 | 0 | 0 | 20 |
|  | 235 | 20 | 0 | 0 | 0 | 20 |
|  | 236 | 20 | 0 | 0 | 0 | 20 |
|  | 237 | 20 | 0 | 0 | 0 | 20 |
|  | 238 | 20 | 0 | 0 | 0 | 20 |
|  | 239 | 20 | 0 | 0 | 0 | 20 |
|  | 240 | 20 | 0 | 0 | 0 | 20 |
|  | 241 | 0 | 20 | 0 | 0 | 20 |
|  | 242 | 0 | 20 | 0 | 0 | 20 |
|  | 243 | 0 | 20 | 0 | 0 | 20 |
|  | 244 | 0 | 20 | 0 | 0 | 20 |
|  | 245 | 0 | 20 | 0 | 0 | 20 |
|  | 246 | 0 | 20 | 0 | 0 | 20 |
|  | 247 | 0 | 20 | 0 | 0 | 20 |
|  | 248 | 0 | 20 | 0 | 0 | 20 |
|  | 249 | 0 | 20 | 0 | 0 | 20 |
|  | 250 | 0 | 20 | 0 | 0 | 20 |
|  | 251 | 0 | 20 | 0 | 0 | 20 |
|  | 252 | 0 | 20 | 0 | 0 | 20 |
|  | 253 | 0 | 20 | 0 | 0 | 20 |
|  | 254 | 0 | 20 | 0 | 0 | 20 |
|  | 255 | 0 | 20 | 0 | 0 | 20 |
|  | 256 | 0 | 20 | 0 | 0 | 20 |
|  | 257 | 0 | 20 | 0 | 0 | 20 |
|  | 258 | 0 | 20 | 0 | 0 | 20 |
|  | 259 | 0 | 20 | 0 | 0 | 20 |
|  | 260 | 0 | 20 | 0 | 0 | 20 |
|  | 261 | 0 | 20 | 0 | 0 | 20 |
|  | 262 | 0 | 20 | 0 | 0 | 20 |
|  | 263 | 0 | 20 | 0 | 0 | 20 |
|  | 264 | 0 | 20 | 0 | 0 | 20 |
|  | 265 | 0 | 20 | 0 | 0 | 20 |
|  | 266 | 0 | 20 | 0 | 0 | 20 |
|  | 267 | 0 | 20 | 0 | 0 | 20 |
|  | 268 | 0 | 20 | 0 | 0 | 20 |
|  | 269 | 0 | 20 | 0 | 0 | 20 |
|  | 270 | 0 | 20 | 0 | 0 | 20 |
|  | 271 | 0 | 0 | 0 | 20 | 20 |
|  | 272 | 0 | 0 | 0 | 20 | 20 |
|  | 273 | 0 | 0 | 0 | 20 | 20 |
|  | 274 | 0 | 0 | 0 | 20 | 20 |
|  | 275 | 0 | 0 | 0 | 20 | 20 |
|  | 276 | 0 | 0 | 0 | 20 | 20 |
|  | 277 | 0 | 0 | 0 | 20 | 20 |
|  | 278 | 0 | 0 | 0 | 20 | 20 |
|  | 279 | 0 | 0 | 0 | 20 | 20 |
|  | 280 | 0 | 0 | 0 | 20 | 20 |
|  | 281 | 0 | 0 | 0 | 20 | 20 |
|  | 282 | 0 | 0 | 0 | 20 | 20 |
|  | 283 | 0 | 0 | 0 | 20 | 20 |
|  | 284 | 0 | 0 | 0 | 20 | 20 |
|  | 285 | 0 | 0 | 0 | 20 | 20 |
|  | 286 | 0 | 0 | 0 | 20 | 20 |
|  | 287 | 0 | 0 | 0 | 20 | 20 |
|  | 288 | 0 | 0 | 0 | 20 | 20 |
|  | 289 | 0 | 0 | 0 | 20 | 20 |
|  | 290 | 0 | 0 | 0 | 20 | 20 |
|  | 291 | 0 | 0 | 0 | 20 | 20 |
|  | 292 | 0 | 0 | 0 | 20 | 20 |
|  | 293 | 0 | 0 | 0 | 20 | 20 |
|  | 294 | 0 | 0 | 0 | 20 | 20 |
|  | 295 | 0 | 0 | 0 | 20 | 20 |
|  | 296 | 0 | 0 | 0 | 20 | 20 |
|  | 297 | 0 | 0 | 0 | 20 | 20 |
|  | 298 | 0 | 0 | 0 | 20 | 20 |
|  | 299 | 0 | 0 | 0 | 20 | 20 |
|  | 300 | 0 | 0 | 0 | 20 | 20 |
| Total | | 1200 | 1201 | 1200 | 1200 | 4801 |

| **Are you currently pregnant * dist_old_intCrosstabulation** | | | | | | |
| --- | --- | --- | --- | --- | --- | --- |
| Count | | | | | | |
|  | | dist_old_int | | | | Total |
|  |  | 1.00 | 2.00 | 3.00 | 4.00 |  |
| Are you currently pregnant | Yes | 57 | 46 | 45 | 75 | 223 |
|  | No | 1143 | 1154 | 1155 | 1125 | 4577 |
| Total | | 1200 | 1200 | 1200 | 1200 | 4800 |

| **resg * dist_old_intCrosstabulation** | | | | | | |
| --- | --- | --- | --- | --- | --- | --- |
| Count | | | | | | |
|  | | dist_old_int | | | | Total |
|  |  | 1.00 | 2.00 | 3.00 | 4.00 |  |
| resg | Mother of under-1 live child | 511 | 461 | 494 | 529 | 1995 |
|  | Mother whose under-1 child died in the past year | 28 | 23 | 20 | 26 | 97 |
|  | Mother who had Abortion/MR/Still birth/IUD | 61 | 116 | 86 | 45 | 308 |
|  | Mother of child aged 12-59 months | 600 | 600 | 600 | 600 | 2400 |
| Total | | 1200 | 1200 | 1200 | 1200 | 4800 |

| **grp1_3 * dist_old_intCrosstabulation** | | | | | | |
| --- | --- | --- | --- | --- | --- | --- |
| Count | | | | | | |
|  | | dist_old_int | | | | Total |
|  |  | 1.00 | 2.00 | 3.00 | 4.00 |  |
| grp1_3 | Abortion | 36 | 73 | 51 | 27 | 187 |
|  | MR | 16 | 33 | 22 | 2 | 73 |
|  | Still Birth | 5 | 7 | 4 | 7 | 23 |
|  | IUD | 4 | 3 | 9 | 9 | 25 |
| Total | | 61 | 116 | 86 | 45 | 308 |

**by adding live births and still births we get total births (last one year)

| **resg * dist_old_intCrosstabulation** | | | | | | |
| --- | --- | --- | --- | --- | --- | --- |
| Count | | | | | | |
|  | | dist_old_int | | | | Total |
|  |  | 1.00 | 2.00 | 3.00 | 4.00 |  |
| resg | Mother of under-1 live child | 511 | 461 | 494 | 529 | 1995 |
|  | Mother whose under-1 child died in the past year | 28 | 23 | 20 | 26 | 97 |
|  | Mother who had Abortion/MR/Still birth/IUD | 61 | 116 | 86 | 45 | 308 |
|  | Mother of child aged 12-59 months | 600 | 600 | 600 | 600 | 2400 |
| Total | | 1200 | 1200 | 1200 | 1200 | 4800 |

Infant mortality =$\frac{total infant death in last one year}{total live births in last one year}$*1000

| **Have Electricity in your household? * dist_old_intCrosstabulation** | | | | | | | |
| --- | --- | --- | --- | --- | --- | --- | --- |
|  | | | dist_old_int | | | | Total |
|  |  |  | 1.00 | 2.00 | 3.00 | 4.00 |  |
| Have Electricity in your household? | Yes | Count | 248 | 353 | 245 | 407 | 1253 |
|  |  | % within dist_old_int | 20.7% | 29.4% | 20.4% | 33.9% | 26.1% |
|  | No | Count | 952 | 847 | 955 | 793 | 3547 |
|  |  | % within dist_old_int | 79.3% | 70.6% | 79.6% | 66.1% | 73.9% |
| Total | | Count | 1200 | 1200 | 1200 | 1200 | 4800 |
|  |  | % within dist_old_int | 100.0% | 100.0% | 100.0% | 100.0% | 100.0% |

| **puca_floor_wall * dist_old_intCrosstabulation** | | | | | | | |
| --- | --- | --- | --- | --- | --- | --- | --- |
|  | | | dist_old_int | | | | Total |
|  |  |  | 1.00 | 2.00 | 3.00 | 4.00 |  |
| puca_floor_wall | .00 | Count | 1159 | 1131 | 1154 | 1167 | 4611 |
|  |  | % within dist_old_int | 96.6% | 94.2% | 96.2% | 97.2% | 96.0% |
|  | 1.00 | Count | 41 | 70 | 46 | 33 | 190 |
|  |  | % within dist_old_int | 3.4% | 5.8% | 3.8% | 2.8% | 4.0% |
| Total | | Count | 1200 | 1201 | 1200 | 1200 | 4801 |
|  |  | % within dist_old_int | 100.0% | 100.0% | 100.0% | 100.0% | 100.0% |

| **wood_fuel * dist_old_intCrosstabulation** | | | | | | | |
| --- | --- | --- | --- | --- | --- | --- | --- |
|  | | | dist_old_int | | | | Total |
|  |  |  | 1.00 | 2.00 | 3.00 | 4.00 |  |
| wood_fuel | .00 | Count | 923 | 965 | 944 | 548 | 3380 |
|  |  | % within dist_old_int | 76.9% | 80.3% | 78.7% | 45.7% | 70.4% |
|  | 1.00 | Count | 277 | 236 | 256 | 652 | 1421 |
|  |  | % within dist_old_int | 23.1% | 19.7% | 21.3% | 54.3% | 29.6% |
| Total | | Count | 1200 | 1201 | 1200 | 1200 | 4801 |
|  |  | % within dist_old_int | 100.0% | 100.0% | 100.0% | 100.0% | 100.0% |

| **sanitary_latrine * dist_old_intCrosstabulation** | | | | | | | |
| --- | --- | --- | --- | --- | --- | --- | --- |
|  | | | dist_old_int | | | | Total |
|  |  |  | 1.00 | 2.00 | 3.00 | 4.00 |  |
| sanitary_latrine | .00 | Count | 921 | 1040 | 1033 | 1059 | 4053 |
|  |  | % within dist_old_int | 76.8% | 86.6% | 86.1% | 88.2% | 84.4% |
|  | 1.00 | Count | 279 | 161 | 167 | 141 | 748 |
|  |  | % within dist_old_int | 23.2% | 13.4% | 13.9% | 11.8% | 15.6% |
| Total | | Count | 1200 | 1201 | 1200 | 1200 | 4801 |
|  |  | % within dist_old_int | 100.0% | 100.0% | 100.0% | 100.0% | 100.0% |

| **Drinking water recoded * dist_old_intCrosstabulation** | | | | | | | |
| --- | --- | --- | --- | --- | --- | --- | --- |
|  | | | dist_old_int | | | | Total |
|  |  |  | 1.00 | 2.00 | 3.00 | 4.00 |  |
| Drinking water recoded | Tubewell | Count | 1175 | 1177 | 1196 | 1174 | 4722 |
|  |  | % within dist_old_int | 97.9% | 98.1% | 99.7% | 97.8% | 98.4% |
|  | Others | Count | 25 | 23 | 4 | 26 | 78 |
|  |  | % within dist_old_int | 2.1% | 1.9% | 0.3% | 2.2% | 1.6% |
| Total | | Count | 1200 | 1200 | 1200 | 1200 | 4800 |
|  |  | % within dist_old_int | 100.0% | 100.0% | 100.0% | 100.0% | 100.0% |

| **soap_used * dist_old_intCrosstabulation** | | | | | | | |
| --- | --- | --- | --- | --- | --- | --- | --- |
|  | | | dist_old_int | | | | Total |
|  |  |  | 1.00 | 2.00 | 3.00 | 4.00 |  |
| soap_used | .00 | Count | 525 | 538 | 731 | 704 | 2498 |
|  |  | % within dist_old_int | 43.8% | 44.8% | 60.9% | 58.7% | 52.0% |
|  | 1.00 | Count | 675 | 663 | 469 | 496 | 2303 |
|  |  | % within dist_old_int | 56.2% | 55.2% | 39.1% | 41.3% | 48.0% |
| Total | | Count | 1200 | 1201 | 1200 | 1200 | 4801 |
|  |  | % within dist_old_int | 100.0% | 100.0% | 100.0% | 100.0% | 100.0% |

****All are similar in table 2 (only for endline)

**SB Table. Percentage of ever had abortion**

| **Ever had abortion * dist_old_endCrosstabulation** | | | | | |
| --- | --- | --- | --- | --- | --- |
|  | | | dist_old_end | | Total |
|  |  |  | intervention_end | control_end |  |
| Ever had abortion | 1 | Count | 441 | 246 | 687 |
|  |  | % within dist_old_end | 13.8% | 15.4% | 14.3% |
|  | 2 | Count | 2759 | 1354 | 4113 |
|  |  | % within dist_old_end | 86.2% | 84.6% | 85.7% |
| Total | | Count | 3200 | 1600 | 4800 |
|  |  | % within dist_old_end | 100.0% | 100.0% | 100.0% |

| **Ever had MR * dist_old_endCrosstabulation** | | | | | |
| --- | --- | --- | --- | --- | --- |
|  | | | dist_old_end | | Total |
|  |  |  | intervention_end | control_end |  |
| Ever had MR | 1 | Count | 123 | 48 | 171 |
|  |  | % within dist_old_end | 3.8% | 3.0% | 3.6% |
|  | 2 | Count | 3077 | 1552 | 4629 |
|  |  | % within dist_old_end | 96.2% | 97.0% | 96.4% |
| Total | | Count | 3200 | 1600 | 4800 |
|  |  | % within dist_old_end | 100.0% | 100.0% | 100.0% |

| **currently used FP method * dist_old_endCrosstabulation** | | | | | |
| --- | --- | --- | --- | --- | --- |
|  | | | dist_old_end | | Total |
|  |  |  | intervention_end | control_end |  |
| currently used FP method | Yes | Count | 2208 | 1056 | 3264 |
|  |  | % within dist_old_end | 69.0% | 66.0% | 68.0% |
|  | No | Count | 992 | 544 | 1536 |
|  |  | % within dist_old_end | 31.0% | 34.0% | 32.0% |
| Total | | Count | 3200 | 1600 | 4800 |
|  |  | % within dist_old_end | 100.0% | 100.0% | 100.0% |

| **Using modern contraceptive method * dist_old_endCrosstabulation** | | | | | |
| --- | --- | --- | --- | --- | --- |
|  | | | dist_old_end | | Total |
|  |  |  | intervention_end | control_end |  |
| Using modern contraceptive method | No | Count | 1160 | 623 | 1783 |
|  |  | % within dist_old_end | 36.2% | 38.9% | 37.1% |
|  | Yes | Count | 2040 | 977 | 3017 |
|  |  | % within dist_old_end | 63.8% | 61.1% | 62.9% |
| Total | | Count | 3200 | 1600 | 4800 |
|  |  | % within dist_old_end | 100.0% | 100.0% | 100.0% |

| **re_Preg_iden*dist_old_endCrosstabulation** | | | | | |
| --- | --- | --- | --- | --- | --- |
|  | | | dist_old_end | | Total |
|  |  |  | intervention_end | control_end |  |
| re pregnancy identification^a^ | Self | Count | 798 | 508 | 1306 |
|  |  | % within dist_old_end | 49.9% | 63.5% |  |
|  | RBAC CHW | Count | 371 | 14 | 385 |
|  |  | % within dist_old_end | 23.2% | 1.8% |  |
|  | Other professionals | Count | 266 | 170 | 711 |
|  |  | % within dist_old_end | 16.6% | 21.2% |  |
| Total | | Count | 1600 | 800 | 2400 |
| Percentages and totals are based on respondents. | | | | | |
| a. Group | | | | | |

| **ANC check up during last pregnancy * dist_old_endCrosstabulation** | | | | | |
| --- | --- | --- | --- | --- | --- |
|  | | | dist_old_end | | Total |
|  |  |  | intervention_end | control_end |  |
| ANC check up during last pregnancy | Yes | Count | 1427 | 553 | 1980 |
|  |  | % within dist_old_end | 89.2% | 69.1% | 82.5% |
|  | No | Count | 173 | 247 | 420 |
|  |  | % within dist_old_end | 10.8% | 30.9% | 17.5% |
| Total | | Count | 1600 | 800 | 2400 |
|  |  | % within dist_old_end | 100.0% | 100.0% | 100.0% |

| **take tt during last pregnancy * dist_old_endCrosstabulation** | | | | | |
| --- | --- | --- | --- | --- | --- |
|  | | | dist_old_end | | Total |
|  |  |  | intervention_end | control_end |  |
| take tt during last pregnancy | Yes | Count | 806 | 319 | 1125 |
|  |  | % within dist_old_end | 50.4% | 39.9% | 46.9% |
|  | No | Count | 202 | 79 | 281 |
|  |  | % within dist_old_end | 12.6% | 9.9% | 11.7% |
|  | Had completed dose earlier | Count | 588 | 401 | 989 |
|  |  | % within dist_old_end | 36.8% | 50.1% | 41.2% |
|  | Don't remember | Count | 4 | 1 | 5 |
|  |  | % within dist_old_end | 0.2% | 0.1% | 0.2% |
| Total | | Count | 1600 | 800 | 2400 |
|  |  | % within dist_old_end | 100.0% | 100.0% | 100.0% |

| **Place of delivery recoded * dist_old_endCrosstabulation** | | | | | |
| --- | --- | --- | --- | --- | --- |
|  | | | dist_old_end | | Total |
|  |  |  | intervention_end | control_end |  |
| Place of delivery recoded | SYS | Count | 223 | 119 | 342 |
|  |  | % within dist_old_end | 13.9% | 14.9% | 14.2% |
|  | Home | Count | 980 | 462 | 1442 |
|  |  | % within dist_old_end | 61.2% | 57.8% | 60.1% |
|  | On the way | Count | 8 | 5 | 13 |
|  |  | % within dist_old_end | 0.5% | 0.6% | 0.5% |
|  | Public facility | Count | 228 | 82 | 310 |
|  |  | % within dist_old_end | 14.2% | 10.2% | 12.9% |
|  | Private or other facility | Count | 161 | 132 | 293 |
|  |  | % within dist_old_end | 10.1% | 16.5% | 12.2% |
| Total | | Count | 1600 | 800 | 2400 |
|  |  | % within dist_old_end | 100.0% | 100.0% | 100.0% |

| **Attendant at delivery * dist_old_endCrosstabulation** | | | | | |
| --- | --- | --- | --- | --- | --- |
|  | | | dist_old_end | | Total |
|  |  |  | intervention_end | control_end |  |
| Attendant at delivery | .00 | Count | 223 | 119 | 342 |
|  |  | % within dist_old_end | 13.9% | 14.9% | 14.2% |
|  | SBA | Count | 448 | 219 | 667 |
|  |  | % within dist_old_end | 28.0% | 27.4% | 27.8% |
|  | not_SBA | Count | 929 | 462 | 1391 |
|  |  | % within dist_old_end | 58.1% | 57.8% | 58.0% |
| Total | | Count | 1600 | 800 | 2400 |
|  |  | % within dist_old_end | 100.0% | 100.0% | 100.0% |

| **cord_strailed * new_dist_old_endCrosstabulation** | | | | | |
| --- | --- | --- | --- | --- | --- |
|  | | | new_dist_old_end | | Total |
|  |  |  | intervention_end | control_end |  |
| cord_strailed | .00 | Count | 87 | 97 | 184 |
|  |  | % within new_dist_old_end | 6.4% | 14.5% | 9.1% |
|  | 1.00 | Count | 1271 | 574 | 1845 |
|  |  | % within new_dist_old_end | 93.6% | 85.5% | 90.9% |
| Total | | Count | 1358 | 671 | 2029 |
|  |  | % within new_dist_old_end | 100.0% | 100.0% | 100.0% |

| **Bathing on within 6 hours * new_dist_old_endCrosstabulation** | | | | | |
| --- | --- | --- | --- | --- | --- |
|  | | | new_dist_old_end | | Total |
|  |  |  | intervention_end | control_end |  |
| Bathing on within 6 hours | No | Count | 1225 | 593 | 1818 |
|  |  | % within new_dist_old_end | 90.2% | 88.4% | 89.6% |
|  | Yes | Count | 133 | 78 | 211 |
|  |  | % within new_dist_old_end | 9.8% | 11.6% | 10.4% |
| Total | | Count | 1358 | 671 | 2029 |
|  |  | % within new_dist_old_end | 100.0% | 100.0% | 100.0% |

| **col_bres * new_dist_old_endCrosstabulation** | | | | | |
| --- | --- | --- | --- | --- | --- |
|  | | | new_dist_old_end | | Total |
|  |  |  | intervention_end | control_end |  |
| col_bres | .00 | Count | 277 | 144 | 421 |
|  |  | % within new_dist_old_end | 20.4% | 21.5% | 20.7% |
|  | 1.00 | Count | 1081 | 527 | 1608 |
|  |  | % within new_dist_old_end | 79.6% | 78.5% | 79.3% |
| Total | | Count | 1358 | 671 | 2029 |
|  |  | % within new_dist_old_end | 100.0% | 100.0% | 100.0% |

| **Exclusively breastfed up to 6 months * new_dist_old_endCrosstabulation** | | | | | |
| --- | --- | --- | --- | --- | --- |
|  | | | new_dist_old_end | | Total |
|  |  |  | intervention_end | control_end |  |
| Exclusively breastfed up to 6 months | No | Count | 890 | 494 | 1384 |
|  |  | % within new_dist_old_end | 65.5% | 73.6% | 68.2% |
|  | Yes | Count | 468 | 177 | 645 |
|  |  | % within new_dist_old_end | 34.5% | 26.4% | 31.8% |
| Total | | Count | 1358 | 671 | 2029 |
|  |  | % within new_dist_old_end | 100.0% | 100.0% | 100.0% |

**SC Table. Distribution of home and facility deliveries**

| **Place of delivery recoded * year Crosstabulation** | | | | | | |
| --- | --- | --- | --- | --- | --- | --- |
|  | | | year | | | Total |
|  |  |  | 2008 | 2010 | 2012 |  |
| Place of delivery recoded | Home | Count | 1784 | 1169 | 980 | 3933 |
|  |  | % within year | 84.3% | 78.6% | 71.2% | 79.0% |
|  | On the way | Count | 10 | 6 | 8 | 24 |
|  |  | % within year | 0.5% | 0.4% | 0.6% | 0.5% |
|  | Public facility | Count | 187 | 204 | 228 | 619 |
|  |  | % within year | 8.8% | 13.7% | 16.6% | 12.4% |
|  | Private or other facility | Count | 134 | 109 | 161 | 404 |
|  |  | % within year | 6.3% | 7.3% | 11.7% | 8.1% |
| Total | | Count | 2115 | 1488 | 1377 | 4980 |
|  |  | % within year | 100.0% | 100.0% | 100.0% | 100.0% |
